# Chinese herbal medicine research in eczema treatment

**DOI:** 10.1186/1749-8546-6-17

**Published:** 2011-04-28

**Authors:** Kam Lun Hon, Ben Chung-Lap Chan, Ping Chung Leung

**Affiliations:** 1 Departments of Paediatrics, The Chinese University of Hong Kong, Prince of Wales Hospital, Shatin, Hong Kong SAR, China; 2 Institute of Chinese Medicine, The Chinese University of Hong Kong, Prince of Wales Hospital, Shatin, Hong Kong SAR, China

## Abstract

Eczema is a chronic relapsing atopic dermatitis (AD) associated with pruritus, sleep disturbance and poor quality of life of the patient. Treatment of eczema includes use of emollient, topical and systemic antimicrobial agents, corticosteroid or immunomodulating agents. Many patients also seek alternative treatments such as dietary avoidance, supplementation or both. This article reviews the basic pathophysiology of eczema and clinical trials involving Chinese medicine in the treatment of eczema. Research reports on Chinese herbal medicine for eczema were retrieved from PubMed and the Cochrane Database for Systematic Reviews for this review. Only a few RCTs demonstrated the efficacy (or lack of efficacy) of Chinese medicinal herbs in treating atopic eczema. Further larger scale trials are warranted.

## Introduction

Atopic dermatitis (AD) is a chronically relapsing inflammatory skin disease commonly associated with allergy [1,2]. About 15% of children suffer from this disease [2-4]. Typical onset of the disease is occurs in the children under five years of age [2,5]. The condition improves in most patients before adulthood. Management of this condition includes use of emollient, topical and systemic antimicrobial agents, corticosteroid or immunomodulating agents [2]. Corticosteroids (CS) are the common treatment for AD in either topical or systemic form. CS has a wide range of immunomodulatory effects, such as the suppression of cytokine production, adhesion molecule expression and leukocyte chemotaxis [6]. CS is also associated with deranged metabolism, growth suppression and increased susceptibility to infections. In particular, the use of potent topical CS in AD may cause significant suppression of the hypothalamic-pituitary-adrenal axis [7]. More specific immunomodulatory agents (*eg *topical tacrolimus) are available [2,8]. As there is still no cure for AD, various dietary therapies including Chinese medicine are adopted by the patients, especially in Asia [9]. However, the beneficial effects of Chinese medicine on children with AD have not been consistently demonstrated [10]. A limited number of Chinese medicine trials in children and adults with AD did not show convincing results [11-14].

## Pathogenesis

Pathogenesis of AD involves complex interactions between susceptible genes (filaggrin genes), immunological factors (immunoglobulin E, eosinophils, T helper cells, chemokines), skin barrier defects, infections, neuroendocrine factors (brain derived neurotrophic factor) and environmental factors (weather change, food and aeroallergens) [1,2,15,16]. Major components in immune dysregulation include Langerhans' cells, inflammatory dendritic epidermal cells, monocytes, macrophages, lymphocytes, mast cells and keratinocytes. All of these components interact through an intricate cascade of cytokines leading to a predominance of Th2 cells [16]. Th2 cytokines, interleukins IL-4, IL-5, IL-10 and IL-13, increase in the skin while there is a corresponding decrease in Th1 cytokines, mainly interferon-γ and IL-2 [2].

Changes in the epidermis are attributed to the xerotic skin in AD patient. Essential fatty acids (EFAs) are important components of the epidermis. Loss of EFAs results in increased transepidermal water loss and subsequent xerosis (dryness). Defects in the epidermal barrier also lead to increased susceptibility to allergens such as house dust mites, grass or pollen. When such allergens are in contact with susceptible skin, they stimulate Th2 lymphocytes to produce cytokines such as IL-4, IL-5 and IL-13 which in turn promote an increase in IgE synthesis [2,17,18]. AD patients often have high levels of IgE antibodies in response to house dust mites and other allergens [19,20].

AD patients also often have defective cell-mediated immunity, which is attributed to increased susceptibility to many bacterial, viral and fungal infections of the skin [2]. Certain factors, including *Staphylococcus aureus *colonization, stress, anxiety, systemic illness and xerosis, exacerbate or trigger AD [2].

According to Chinese medicine theory [21], Qi can be disrupted by 'wind', 'coldness', 'summer-heat', 'dampness', 'dryness' or 'fire evils'. Main pathogenic factors of eczema are thought to be 'wind', 'dampness' and 'heat' [21]. Herbs such as *Cortex Moutan Radix *(*Danpi*), *Radix Paeoniae Alba *(*Bai Shao*), *Potentilla Chinensis Ser *(*Weilingcai) *and *Radix Glycyrrhizae *(*Gan Cao*) are common treatments for allergy [22]. *Flos Lonicerae *(*Jingyinhua*) and *Herba Menthae *(*Bohe*) clear 'damp-heat' from the exterior, *Cortex Moutan *(*Danpi*) clears 'heat' from blood while *Rhizoma Atractylodis *(*Cangzhu*) and *Cortex Phellodendri *(*Huangbai*) clear the 'damp-heat' from the interior. Pharmacological studies indicate that these herbs have anti-allergic, anti-inflammatory and sedative action for itchiness [21,23,24].

This article aims to review randomized trials, case series and bench studies in Chinese medicine for eczema.

For this review, as of December 2010, we retrieved 47 articles from PubMed using the keywords "'Chinese herbal medicine' and ('atopic dermatitis' or 'eczema')". We also searched the Cochrane Database for Systematic Reviews. Using PubMed Clinical Queries, we retrieved 9, 2 and 9 references under Clinical Study Categories, Systematic Reviews and Medical Genetics respectively. All RCTs and relevant case series and bench studies were included. Review articles that did not provide any information on eczema and Chinese medicine were excluded.

## Randomized trials

There have been only a few randomized trials in this region on Chinese medicine treatment for AD (Table 1). Cochrane systematic review in 2005 on the topic of Chinese herbal medicine for AD included only four clinical trials [13].

**Table 1:** Randomized trials on herbal medicine for eczema

Population	Design	Outcome	Adverse effects	Validity	Remarks	
Pediatric	Randomized, double-blind, placebo-controlled, crossover	Clinical only	Nil	Not intention-to-treat due to dropouts, no quality of life measurement	Efficacy not concurred with [11]	[25]
Adults	Randomized, double-blind, placebo-controlled, crossover	Clinical only	Nil	Not intention-to-treat due to dropouts, no quality of life measurement	Efficacy not concurred with [11]	[13,49]
Pediatric + Adults	Randomized, double-blind, placebo-controlled, crossover	Clinical only	Minor	Intention-to-treat, no dropouts, no quality of life measurement	No effects	[11]
Pediatric	Randomized, double-blind, placebo-controlled	Clinical + quality of life + oral antihistamine and topical steroid-sparing	Minor	Intention-to-treat, no dropouts, quality of life + oral antihistamine and topical steroid usage sparing	Improved quality of life and steroid-sparing	[30]
Adults	Randomized to 4 groups, saline as control	Poorly defined total effective rate and cured rate	Nil	Apparently no dropouts, but very small subgroups sizes, no quality of life measurement	Effective, difficult to assess efficacy	[31]
Adults	Randomized, double-blind, placebo-controlled	Clinical + topical steroid and tacrolimus-sparing	Minor	Not intention-to-treat due to dropouts, no quality of life measurement	Topical steroid and tacrolimus-sparing only	[32]

### Zemphyte trials

In the early 1990s, a decoction (Zemaphyte, Phytopharm Plc, UK) was efficacious for the treatment of AD in both children [25,26] and adults [27] in the UK. Sheehan *et al*. carried out a randomized placebo-controlled double-blind trial of a specific prescription formulated for widespread non-exudative atopic eczema [25]. Forty-seven children were given active treatment and placebo in random order, each for eight weeks, with an intervening 4-week wash-out period. Thirty-seven children received all the treatment and completed the study. Active treatment was more effective than the placebo. There was no evidence of hematological, renal or hepatic toxicity. The authors concluded that the Chinese medicinal herbs under their trial have a therapeutic potential in treating eczema and other skin diseases.

The opportunity to continue treatment was offered to the parents of 37 children who had completed a double-blind placebo-controlled trial of the same formulation of Chinese medicinal herbs for atopic eczema [26]. The parents selected continued treatment in all cases, and the progress of the children was monitored for 12 months. The aim was to reinforce the clinical improvement and to reduce treatment frequency progressively. At the end of the 12-month follow-up period, 18 were reported to have at least 90% reduction in eczema activity scores, and five showed moderate improvement. Fourteen children withdrew from the study. Seven of the children discontinued treatment without relapse; the other 16 required treatment to maintain control of their eczema, but only four of these still required daily treatment. Asymptomatic elevation of serum aspartate aminotransferase (AST) to 7-14 times normal values was noted on one occasion in two children whose eczema after treatment was so well that the therapy was stopped. Liver function became normal after eight weeks. The authors suggested that these Chinese medicinal herbs provided a therapeutic option for children with the extensive atopic eczema which did not respond to other treatments.

The adult patients with severe atopic eczema who had completed a similar double-blind placebo-controlled crossover trial were offered continued treatment for one year [27]. Out of the 31 patients who completed the placebo-controlled study and after a washout period and further treatment, 17 continued treatment (group 1), 11 chose not to continue treatment (group 2), one was lost to follow-up and two patients continued treatment but finally decided to stop treatment. At the end of the 12-month period, 12 patients in group 1 had reduction greater than 90% and the remaining five had reduction greater than 60% in clinical scores from baseline values. Clinical scores of patients in group 2 gradually deteriorated during the year. Difference between groups 1 and 2 in clinical scores was highly significant (*P *= 0.005 and *P *= 0.002 for erythema and surface damage respectively). At the end of the 12-month period, no patient in group 1 discontinued treatment although eight patients were on an alternate-day regimen by six months and remained on this regimen until the end of the 12-month period, and seven were able to control their eczema with treatment throughout the period. The remaining two patients continued on daily treatments. Toxicology screening revealed no abnormalities in either blood counts or biochemical parameters in all patients under continued treatment. Improvement in disease was not associated with any significant change in the levels of serum IgE or peripheral blood lymphocyte subsets.

Despite the efficacy reported in the UK trials, a subsequent randomized placebo-controlled, cross-over trial (RCT) of the same decoction in Hong Kong failed to demonstrate beneficial effects on Chinese patients with recalcitrant AD in Hong Kong [11]. Forty recruited patients were given Zemaphyte and placebo in random order, each for eight consecutive weeks with a 4-week wash-out period in between. Scores based on the severity and extent of four clinical parameters (erythema, surface damage, lichenification and scaling) were recorded at baseline and at 4-weekly intervals throughout the 20-week trial period. Thirty-seven patients completed the trial. There was general clinical improvement throughout the trial period in both patient groups, irrespective of whether they received Zemaphyte or placebo first. Zemaphyte, however, offered no statistically significant treatment effect over placebo for all four clinical parameters, except for lichenification at week 4. There were no significant carry-over effects. The results of blood tests for hematologic, renal and liver functions were all normal throughout the trial. The investigators concluded that Zemaphyte did not benefit the Chinese patients with recalcitrant atopic dermatitis in their study.

### PentaHerbs trials

In a pilot study, Hon *et al*. evaluated the clinical and biochemical effects of a Chinese medicine capsule (PentaHerbs capsule) in children with AD [28]. After a run-in period of four weeks, children old enough to manage oral medication were admitted and their disease severity was evaluated by the SCORing Atopic Dermatitis (SCORAD) index. Blood samples were taken for complete blood count, total and allergen-specific immunoglobulin E (IgE), biochemical studies and inflammatory markers of AD severity [serum cutaneous T cell-attracting chemokine (CTACK), macrophage-derived chemokine (MDC), thymus and activation-regulated chemokine (TARC) and eosinophil cationic protein (ECP)] prior to, and after three months of Chinese medicine use. Three PentaHerbs capsules twice a day were prescribed for four months. Patients were followed up monthly to ensure compliance, and SCORAD scores were obtained at each visit. Five boys and four girls participated in the study. All patients had detectable food or inhalant-specific IgE in serum. There was significant improvement in the overall and component SCORAD scores. There were no significant differences between the pre- and post-treatment values of the serum CTACK, MDC, TARC and ECP levels but CTACK showed a decreasing trend (*P *= 0.069). No clinical or biochemical evidence of any adverse drug reaction was found during the study period. The PentaHerbs capsules were well tolerated by the children and apparent (reduction of disease severity) were noted clinically. The authors concluded that a larger, randomized placebo-controlled study is required to confirm the efficacy of this formulation for AD.

The PentaHerbs capsules were manufactured, packaged and labeled by the Chinese Medicine Industry Development Centre, the Hong Kong Institute of Vocation Education (Hong Kong, China). The composition of each herb in the formulation was standardized. The formula comprised 2 grams of *Flos lonicerae *(*Jinyinhua*), 1 gram of *Herba menthae *(*Bohe*), 2 grams of *Cortex moutan *(*Danpi*), 2 grams of *Rhizoma atractylodis *(*Cangzhu*) and 2 grams of *Cortex phellodendri *(*Huangbai*). The dosage calculation was based on the standard Chinese medicine prescription for children (one bowl of herbal tea) [28] and is equivalent to a daily dose of 20 ml of syrup. This dosage is adequate for children of this age. The syrup was formulated and assessed for its quality and safety according to established procedures [28] under the supervision of the Clinical Trials Section of the Institute of Chinese Medicine (ICM) (Hong Kong, China). In particular, the syrup had been tested for heavy metals, microbial products and residual pesticides; the results met the safety standards of Chinese medicine in Hong Kong. A laboratory study found no corticosteroids in the five constituent herbs [29].

In a subsequent double-blinded randomized placebo-controlled trial, the researchers assessed the efficacy and tolerability of the decoction in children with AD [30]. Following a 2-week run-in period, children with long-standing moderate-to-severe AD were randomized to receive a 12-week treatment with a twice-daily dose of three capsules of either PentaHerbs or placebo. The SCORing of Atopic Dermatitis (SCORAD) score, Children's Dermatology Life Quality Index (CDLQI), allergic rhinitis score, and requirement for topical corticosteroid and oral antihistamine were assessed before and at weeks 4, 8, 12 and 16 of treatment. Adverse events, tolerability, hematological and biochemical parameters were monitored during the study. Eighty-five children with AD were recruited. Over 12 weeks, the mean SCORAD score fell from 58.3 to 49.7 in the PentaHerbs group (*n *= 42; *P *= 0.003) and from 56.9 to 46.9 in the placebo group (*n *= 43; *P *= 0.001). However, there was no significant difference in the scores at the corresponding time points between the two groups. The CDLQI of PentaHerbs-treated group was more significantly improved than that of the placebo group at the end of the 3-month treatment and 4 weeks after stopping therapy (*P *= 0.008 and 0.059 respectively). The total amount of topical corticosteroid used was also significantly reduced by one-third in the PentaHerbs group (*P *= 0.024). The formulation was palatable and well tolerated. No serious adverse effects were observed between the groups. The investigators concluded that the PentaHerbs formulation is efficacious in improving quality of life and reducing topical corticosteroid use in children with moderate-to-severe AD.

### Shuangfujin Trial

Bai *et al*. evaluated the effects and safety of Shuangfujin (SFJ) on acute eczema [31]. One hundred and twenty patients with acute eczema were randomly assigned to four groups of same size, namely the saline group, the boric acid group, the Pifukang lotion group and the SFJ group. After four days of treatment with the respective medicine, the symptom score was remarkably lower in the SFJ group than in the other three groups, score in the saline group was higher than that in the boric acid group and the Pifukang lotion group, and difference between the latter two groups was insignificant. The effective rate and recovery rate were 46.4% and 14.3% in the SFJ group, which were equivalent to those in the Pifukang lotion group and significantly higher than those in the other two groups, and the saline group showed the lowest efficacy. The effects on itchiness in the SFJ and the boric acid group were matched, which was higher than those in the Pifukang lotion group, and the lowest was in the saline group. No skin irritation and other adverse reactions were found.

### Hochu-ekki-to trials

*Hochu-ekki-to*, a Kampo formula (consisting of 10 herbs, namely *Radix Astragali, Panax ginseng C. A. Mey, Rhizoma Atractylodis Macrocephalae, Glycyrrhiza uralensis, Angelica sinensis, Citri Reticulatae Pericarpium, Rhizoma Cimicifugae, Radix Bupleuri, Zingiber officinale Roscoe, Fructus Jujubae Date*), is effective for patients with *Kikyo *(delicate, easily fatigable, or hypersensitive) constitution. Previous case reports suggested that this herbal drug was effective for a subgroup of AD patients. Kobayashi *et al*. evaluated the efficacy and safety of *Hochu-ekki-to *in the long-term management of *Kikyo *patients with AD [32]. In a multicenter, double blind, randomized, placebo-controlled study, 91 *Kikyo *patients with AD were enrolled. *Kikyo *condition was evaluated by a questionnaire scoring system. All patients continued their ordinary treatments (topical steroids, topical tacrolimus, emollients or oral antihistamines) before and after their protocol entry. *Hochu-ekki-to *or placebo was orally administered twice daily for 24 weeks. The skin severity scores, total equivalent amount (TEA) of topical agents used for AD treatment, prominent efficacy (cases with skin severity score = 0 at the end of the study) rate and aggravated rate (more than 50% increase of TEA of topical agents from the beginning of the study) were monitored and evaluated. Seventy-seven out of 91 enrolled patients completed the 24-week treatment course (treatment: *n *= 37, placebo: *n *= 40). The TEA of topical agents (steroids and/or tacrolimus was significantly lower in the *Hochu-ekki-to *group than in the placebo group whereas the overall skin severity scores were not statistically different. The prominent efficacy rate was 19% (7 of 37) in the *Hochu-ekki-to *group and 5% (2 of 40) in the placebo group (*P *= 0.06). The aggravated rate was significantly lower in the *Hochu-ekki-to *group (3%; 1 of 37) than in the placebo group (18%; 7 of 39). Only mild adverse events such as nausea and diarrhea were noted in both groups without statistical difference. This placebo-controlled study demonstrated that *Hochu-ekki-to *was a useful adjunct to conventional treatments for AD patients with *Kikyo *constitution. Use of *Hochu-ekki-to *significantly reduced the dose of topical steroids and/or tacrolimus used for AD treatment without aggravating AD.

## Case series

In the last decades, a number of case series reported the efficacy of herbal medicine on childhood AD. Luo *et al*. reported that fifty-six cases of 'stubborn' ('stubborn' in Chinese probably meant recalcitrance) eczema treated by oral administration and topical application of herbal medicine; [33].

Salameh *et al*. assessed the effectiveness of the combination of Chinese herbal medicine and acupuncture for the treatment of atopic dermatitis [34]. Twenty [20] mild-to-severe atopic dermatitis patients aged between 13 and 48 years were given a combined treatment of acupuncture and Chinese herbal medicine and were followed prospectively. The patients received acupuncture treatment twice a week and the Chinese herbal formula three times daily for a total of 12 weeks. Assessments were performed before treatment, and at weeks 3, 6, 9 and 12 of treatment. The primary outcomes were defined as the changes in the Eczema Area and Severity Index (EASI), Dermatology Life Quality Index (DLQI), and patient assessment of itch measured on a visual analogue scale (VAS). After 12 weeks of treatment, an improvement in EASI over the baseline was noted in 100% of patients. The mean EASI fell from 4.99 to 1.81; the median percentage of decrease was 63.5%. Moreover, 78.8% of patients experienced a reduction in DLQI and VAS, as compared with the baseline. The mean DLQI decreased from 12.5 to 7.6 at the end of treatment, with 39.1% improvement. Mean VAS decreased from 6.8 to 3.7, with 44.7% improvement. No adverse effects were observed. The authors concluded that the combination of acupuncture and Chinese herbal medicine had a beneficial effect (reduction of disease severity and improvement of quality of life) on AD patients, probably better than Chinese herbal medicine alone. As this study involved heterogeneous age groups of both children and adults and it was an open-label study, conclusion about the Chinese herbal medicine cannot be ascertained.

Hon *et al*. performed a single-center open label trial to assess the efficacy and tolerability of a Chinese medicine syrup in younger children with AD [35]. Children aged 4 to 7 years with AD diagnosed according to Hanifin and Rajka's criteria [36] were recruited. The clinical severity of AD was evaluated according to the SCORing Atopic Dermatitis (SCORAD) index, [37] and all subjects had an objective SCORAD score of ≥15 (moderate-to-severe disease) at the entry into this study [38]. At baseline (visit 1), dietary intake, emollient, topical corticosteroid usage and information regarding the severity of AD were collected. They then received Chinese herbal medicine syrup 20 ml daily for 12 weeks. The physicians at the Institute of Chinese Medicine suggested that this dosage (equivalent to a bowel of herbal tea) would suit a wide range of age groups [28,30]. Enrolled subjects were followed up at two weeks (visit 2), seven weeks (visit 3), 12 weeks (visit 4) and four weeks after completion of treatment (visit 5) for the control of their skin condition. Each patient was given an Eczema Diary for recording daily symptom during the period prior to each visit. The severity of AD and quality of life as assessed with the SCORAD index and Children's Dermatology Life Quality Index (CDLQI), respectively [37-39] at every visit were used as the primary outcome measures. The individual components that constitute SCORAD, such as disease extent, intensity, pruritus and sleep loss [37] formed the secondary outcome measures. The potency of topical CS was not changed during the study period. The type of oral anti-histamines used by a particular subject remained the same throughout the study. Twenty-two Chinese children (10 boys and 12 girls), with a mean (Standard deviation, SD) age of 5.8 (0.9) years, participated in this study. Their mean (SD) objective SCORAD was 36.6 (12.5), and mean (SD) CDLQI was 11.9 (6.0). There were significant improvements in the objective SCORAD, pruritus and CDLQI scores four weeks after study completion. There was no change in sleep score or amount of topical steroid consumption. No biochemical evidence of any adverse drug reaction was found during the study period. The Chinese herbal medicine syrup was generally palatable and well tolerated by the children. Adverse effects were mild although two patients with rash withdrew during the study. The authors concluded that further evaluations and dosage studies of the decoction for treating young children were warranted. The findings in quality of life improvement agreed with the previous randomized placebo-controlled trial with the same decoction in capsular preparation for older children [30]. The therapeutic effects persisted one month after treatment had stopped.

## Laboratory studies

To delineate the actions of PentaHerbs on AD, the authors analyzed the effects of an extract of these herbs on interleukin 4 (IL-4)-induced CD23 expression on peripheral blood monocytes collected from non-atopic subjects. They found that PentaHerbs inhibited CD23 expression up to 60% (*P *< 0.001) whereas the placebo extract had no significant effect on CD23 expression. This inhibition was dose-dependent, and PentaHerbs was effective at a concentration of 250 μg/ml (*P *= 0.001). If PentaHerbs or placebo was added after IL-4, the action of PentaHerbs was still observed at 12 hours. This inhibition was not due to cell death, as peripheral blood mononuclear cells (PBMCs) cultured with PentaHerbs or placebo at a concentration used in these experiments had a similar viability to control cultures. Down-regulation of the low affinity receptors for IgE on antigen-presenting cells in patients with eczema may contribute to the benefit observed following treatment with PentaHerbs [40].

Laboratory studies also demonstrated favorable immunomodulatory effects [41,42]. Leung *et al*. investigated the immunomodulatory effects possibly induced by PentaHerbs treatment [41] on cytotoxicity and proliferation of phytohaemagglutinin (PHA)- and staphylococcal enterotoxin B (SEB)-stimulated peripheral blood mononuclear cells (PBMC) isolated from buffy coat of blood donors. PentaHerbs-induced immunomodulation for five inflammatory mediators in cultured PBMC was measured by reverse transcription-polymerase chain reaction (RT-PCR) and enzyme-linked immunosorbent assay. The effects of a 3-month, open-label study of PHF treatment on circulating inflammatory mediators in children with AD were also assessed. PentaHerbs at up to 1 mg/mL dose-dependently suppressed PBMC proliferation. The addition of PentaHerbs to cultured PBMC reduced supernatant concentrations of brain-derived neurotrophic factor (BDNF), interferon (IFN)-gamma and tumour necrosis factor (TNF)-alpha in response to PHA and BDNF and thymus and activation-regulated chemokine (TARC) following SEB stimulation. PentaHerbs increased epithelial cell-derived neutrophil activating peptide-78 levels in culture supernatants. At the RNA level, PentaHerbs suppressed the transcription of BDNF, TARC, IFN-gamma and TNF-alpha. Twenty-eight children with AD were treated with PentaHerbs for three months, and their mean plasma concentrations of BDNF and TARC decreased significantly from 1798 pg/mL and 824 pg/mL at baseline to 1378 pg/mL and 492 pg/mL (*P *= 0.002 and 0.013 respectively) upon study completion. The invetsigators concluded that PentaHerbs possessed *in vitro *and *in vivo *immunomodulation that might mediate the clinical efficacy observed in AD treatment with PentaHerbs [41].

The actions of PentaHerbs on mast cell activation was also investigated [42]. Effects of aqueous extracts of PentaHerbs and individual component herbs on mediator release from rat peritoneal mast cells (RPMCs) and cytokine production from HMC-1 were investigated. PentaHerbs, *Cortex Moutan *and *Herba Menthae *significantly attenuated histamine release and prostaglandin D(2) synthesis from RPMC activated by anti-IgE and compound 48/80. While *Flos Lonicerae *and *Rhizoma Atractylodis *suppressed only mediator release from compound 48/80 activated RPMC, *Cortex Phellodendri *potentiated only anti-IgE induced mediator release. However, with the exception of *Cortex Moutan*, PentaHerbs and the other four component herbs failed to affect cytokine production in Human Mast Cell HMC-1. The investigators concluded that individual herbs of PentaHerbs modulated mast cells and inhibited the inflammatory mediators from mast cells thereby achieving the therapeutic efficacy of PentaHerbs.

## Adverse effects

Adverse effects of some Chinese herbal medicine have been reported [11,43-49]. Perharic *et al*. received reports of 11 cases of liver damage following the use of Chinese herbal medicine for skin conditions [48]. There were two confirmed cases in which recovery after dechallenge and recurrence of hepatitis after rechallenge were observed. The time-course relationship, recovery after ceasing Chinese herbal medicine, and absence of alternative causes of liver damage two further symptomatic cases following a single period of exposure. Herbal material was available for analysis in seven cases. The plant mixtures varied and no single ingredient accounted for the liver injury in this case series. The mechanism of toxicity was unclear; effects did not appear dose-related and are probably idiosyncratic. Ferguson *et al*. reported that a patient with eczema developed a severe cardiomyopathy after a 2-week course of Chinese herbal medicine [44]. The connection between cardiomyopathy and usage of Chinese herbal medicine was not established until when the patient was specifically asked if she had ingested any unusual substances. The authors cautioned that patients might not consider medicinal herbs worth mentioning during a standard medical history. The indiscriminate usage of herbs by parents in treating children for prolonged durations is especially alarming. This concern is increased by the fact that there is no standardized treatment; it is the choice of the individual practitioner how much any one herb is prescribed [43]. Chinese medicine practitioners have another concern that standardisation of herbal mixtures may contradict Chinese medicine theories [43].

## Conclusion

Only a few RCTs demonstrated the efficacy (or lack of efficacy) of Chinese medicinal herbs in treating AD. Further larger scale trials and laboratory studies are warranted.

## Abbreviations

AD: Atopic dermatitis; AST: Aspartate aminotransferase; BDNF: Brain derived neutrotrophic factor; CD: Cluster designation; CDLQI: Children Dermatology Life Quality Index; CS: Corticosteroid; CTACK: Cutaneous T cell-attracting chemokine; DLQI: Dermatology Life Quality Index; EASI: Eczema Area and Severity Index; ECP: Eosinophil cationic protein; EFA: Essential fatty acid; HMC Human mast cell; IFN: Interferon; IL: Interleukin; IgE: Immunoglobulin E; MDC: Macrophage-derived chemokine; PBMCs: Peripheral blood mononuclear cells; PHA: P*hyto*hemagglutinin; RCT: Randomised controlled trial; SCORAD: SCORing Atopic Dermatitis; SD: Standard deviation; SEB: Staphylococcal enterotoxin B; SFJ: Shuangfujin; TARC: Thymus and activation-regulated chemokine; TEA: Total equivalent amount; Th1: T Helper 1; Th2: T Helper 2; TNF: Tumour necrosis factor; UK: United Kingdom; VAS: Visual analogue scale.

## Competing interests

The authors were involved in the design and trials of the PentaHerbs formulation.

## Authors' contributions

KLH drafted the manuscript. BCLC and PCL revised this manuscript. All authors read and approved the final version of the manuscript.
